# International Periodical Congress of Gynæcology and Obstetrics

**Published:** 1892-04

**Authors:** 


### INTERNATIONAL PERIODICAL CONGRESS OF GYNÆCOLOGY AND OBSTETRICS.

The Belgian Society of Gynæcology and Obstetrics, under the patronage of the Belgian Government, has taken the initiative in organizing "The International Periodical Congress of Gynæcology and Obstetrics," the first session of which will be held in Brussels, September 14 to 19 inclusive, 1892. Three leading questions will be offered for discussion:

1st. Pelvic Suppurations; Referee, Dr. Paul Segond, Paris.

2d. Extra Uterine Pregnancy; Referee, Dr. A. Martin, Berlin.

3d. Placenta Prævia; Referee, Dr. Berry Hart, Edinburg.

Fees: Members participating in first session, 30 francs. (This will entitle the holder to a copy of the proceedings of the congress.)

Founders (Life Membership), 300 francs.

In connection with the congress, there will be an international exposition of instruments and appliances, pertaining to gynæcology and obstetrics.

All communications pertaining to this congress should be mailed direct to the American Secretary, Dr. F. Henrotin, 353 La Salle Ave., Chicago, who will promptly furnish all information. All notifications to be forwarded should be received by August 1st.

Everything points to a great success in this congress. Though notices concerning it have been rather late in this country, already men of celebrity have promised to visit and contribute papers. Among the many foreigners who have written to the secretary general endorsing and promising support to the undertaking, may be mentioned the following eminent men:

BELGIUM—De Roubaix, Sacre, Mirriar, Pigeolot, Charles, Sanpart and others.

FRANCE—Pean, Demous, Fochier, Auvard, Doleris, Pozzi, Tarnier, Budin, Terrillon, Terrier and others.

ENGLAND—Lawson Tait, Wm. Priestly, Champneys, G. Elder, J. White, Watt Black, Thornton, Doran, Spencer Wells, Bantock and others.

GERMANY—Martin, Leopold, Sanger, Gusserow, Veit, Winckel, Hegar, Kaltenbach, Freund, Heyder and others.

SWITZERLAND—Reverdin, Vuillet.

RUSSIA—Slaviansky.

SWEDEN—Saliss, Westernark.

NORWAY—Statfeldt, Howitz, Meyer.

ITALY—Porro, La Torre, Mangiazalli, Bozzi, Morisain.

TURKEY—Chatazian.

HOLLAND—Stokvis, Treub, Nyhoff.

AUSTRIA—Pawlik, Albert, Chrobuk.

FINLAND—Engstrom, Heinricius, Pippingohold.

Further details will be furnished as soon as received.

